# Supplementary material for: Tail state limited photocurrent collection of thick photoactive layers in organic solar cells
Source: Nat Commun. 2019 Nov 14;10:5159. doi: 10.1038/s41467-019-12951-7 (PMC6856365; doi:10.1038/s41467-019-12951-7)
Supplement: Supplementary file 1 — Supplementary Information [file 41467_2019_12951_MOESM1_ESM.pdf]

Tail state limited photocurrent collection of thick photoactive layers in organic solar cells

Jiaying Wu[1], *Joel Luke*[2], *Harrison K. H. Lee*[3], *Pabitra Shakya Tuladhar*[1], *Hyojung Cha*[1], *Soo-Young Jang*[1,4], *Wing Chung Tsoi*[3], *Martin Heeney*[1], *Hongkyu Kang*[1,4]*, *Kwanghee Lee*[4], *Thomas Kirchartz*[5,6]*, *Ji-Seon Kim*[2]*, *James R. Durrant*[1,3]*

[1] J. Wu, Dr P. S. Tuladhar, Dr H. Cha. Dr S. Jang, Prof. M. Heeney, Dr H. Kang, Prof. J. R. Durrant

Department of Chemistry and Centre for Plastic Electronics, Imperial College London, London, SW7 2AZ, UK

[2] J. Luke, Prof. J. S. Kim,

Department of Physics and Centre for Plastic Electronics, Imperial College London, London, SW7 2AZ, UK

[3] Dr H. K. H. Lee, Dr W. C. Tsoi, Prof. J. R. Durrant

SPECIFIC, College of Engineering, Bay Campus, Swansea University, Swansea, SA1 8EN, UK

[4] Dr S. Jang, Prof. K. Lee, Dr H. Kang

Research Institute for Solar and Sustainable Energies, Gwangju Institute of Science and Technology, Gwangju 61005, Republic of Korea

[5] Prof. T. Kirchartz

IEK5-Photovoltaik, Forschungszentrum Jülich, 52425 Jülich, Germany

[6] Prof. T. Kirchartz

Faculty of Engineering and CENIDE, University of Duisburg-Essen, Carl-Benz-Strasse 199, 47057 Duisburg, Germany

Emails: j.durrant@imperial.ac.uk, t.kirchartz@fz-juelich.de, ji-seon.kim@imperial.ac.uk, gemk@gist.ac.kr

Supplementary Figures

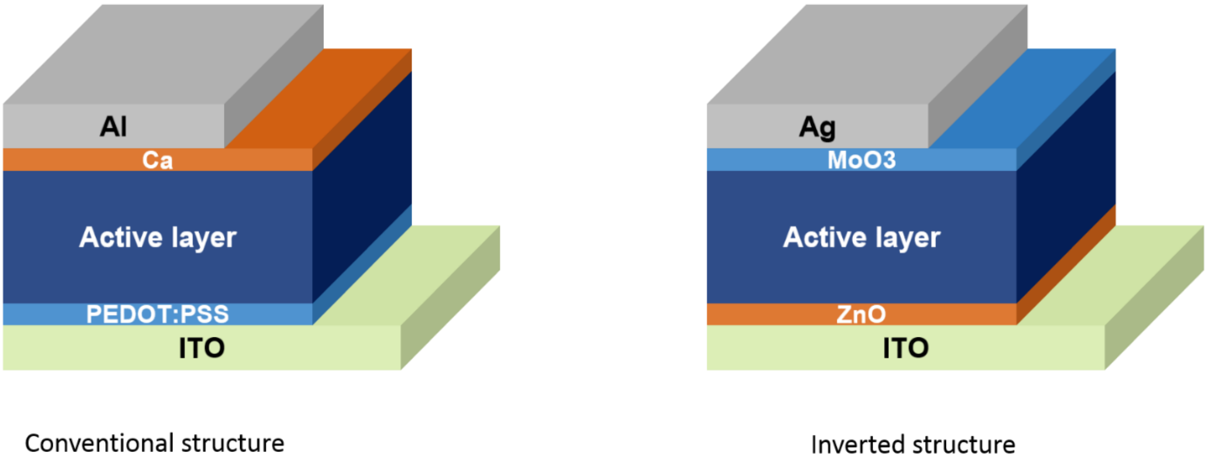

Supplementary Figure 1. Device structures. The BTR small molecular based devices were made using conventional structure, and the rest devices were made using inverted structures.

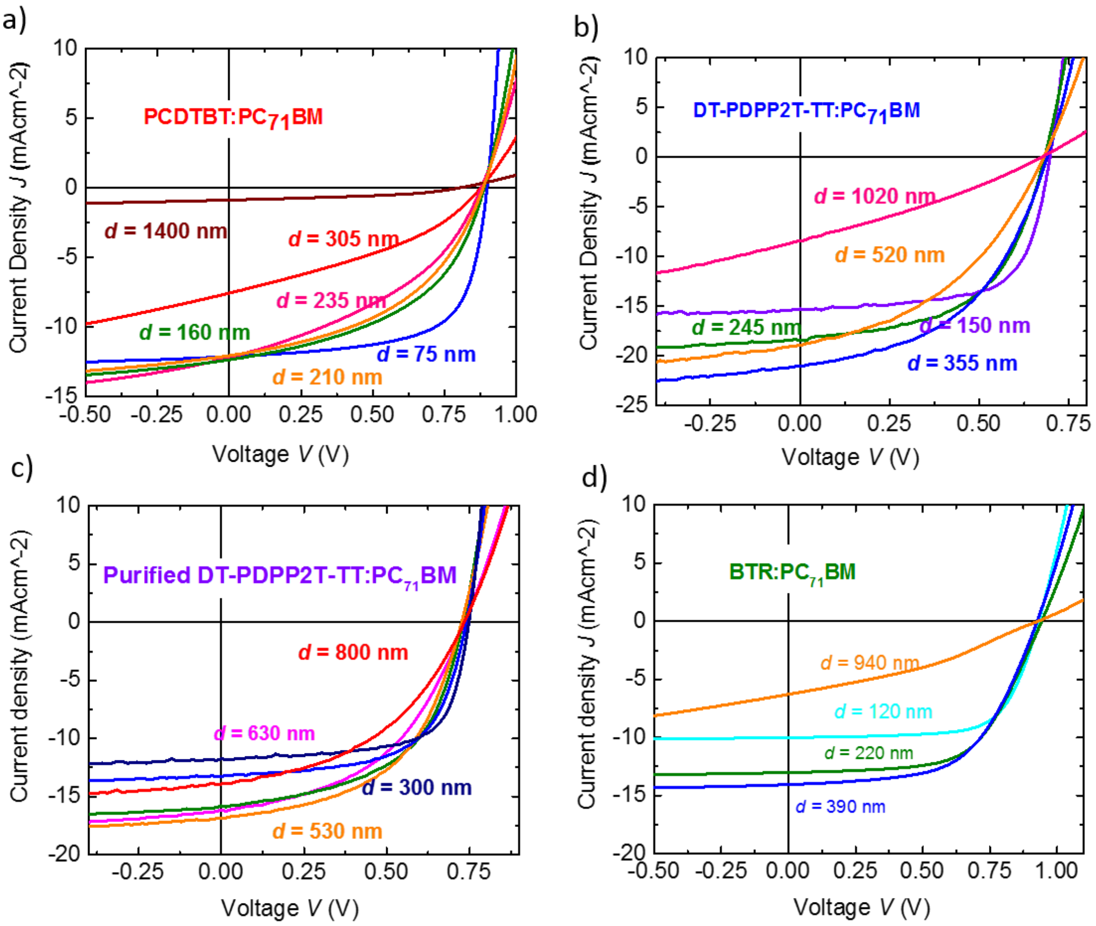

Supplementary Figure 2. Thickness-dependent device *J-V* characterisations. Figure (a), (b), (c) and (d) show the thickness-dependent *J-V* results under standard AM1.5G illumination of PCDDTBT:$PC_{71}BM$, DT-PDPP2T-TT:$PC_{71}BM$, purified-DT-PDPP2T-TT:$PC_{71}BM$ and BTR:$PC_{71}BM$.

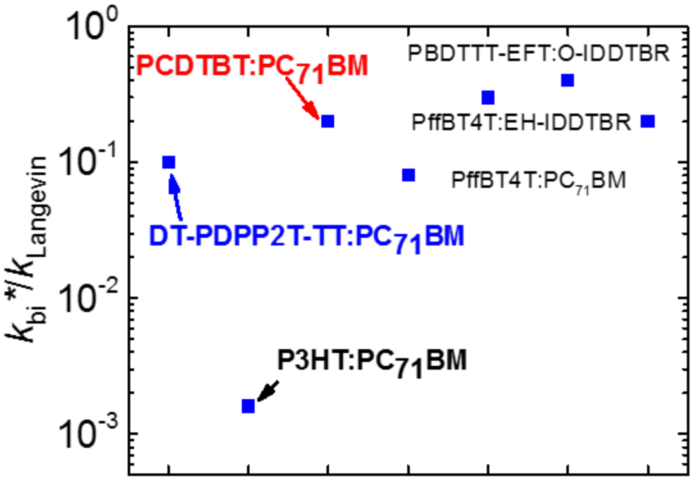

Supplementary Figure 3. Non-Langevin factors of different organic bulk heterojunction devices. Except for P3HT:PC_{71}BM device, the other bulk heterojunction solar cells show relatively Langevin recombination. Non-Langevin factors determined from the quotient of the effective bimolecular recombination coefficient k_{bi} (measured by TPV/CE) over the Langevin recombination coefficient $k_{Langevin}$.

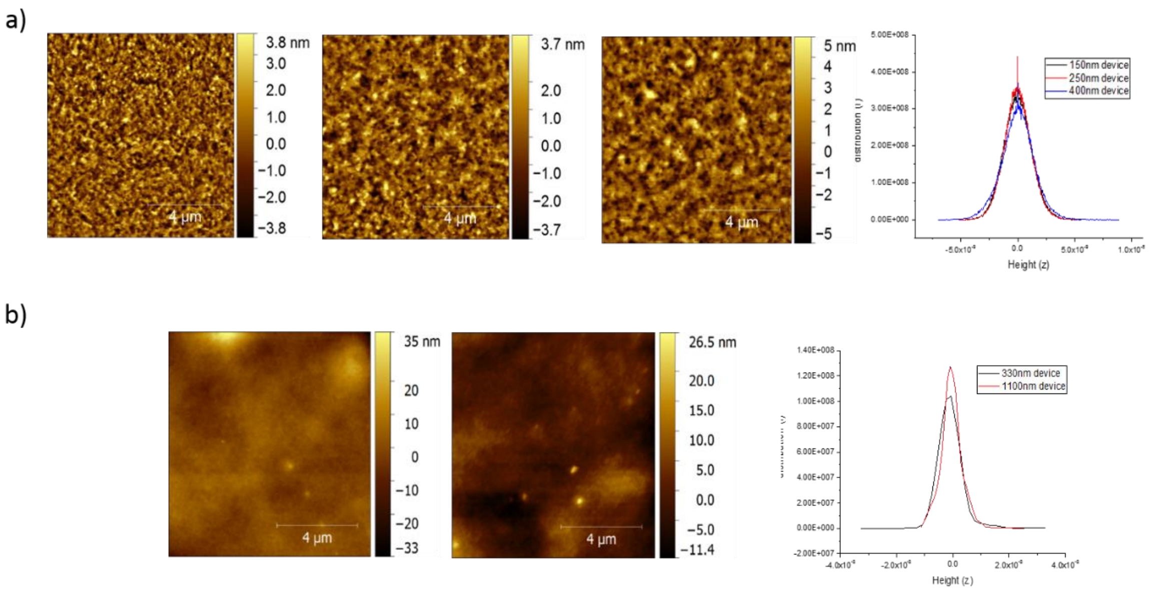

Supplementary Figure 4. AFM results. Figure (a) shows PCDTBT:$PC_{71}BM$ films (150 nm, 250 nm, 400 nm) and (b) DT-PDPP2T-TT:$PC_{71}BM$ films (330 nm, 1100 nm).

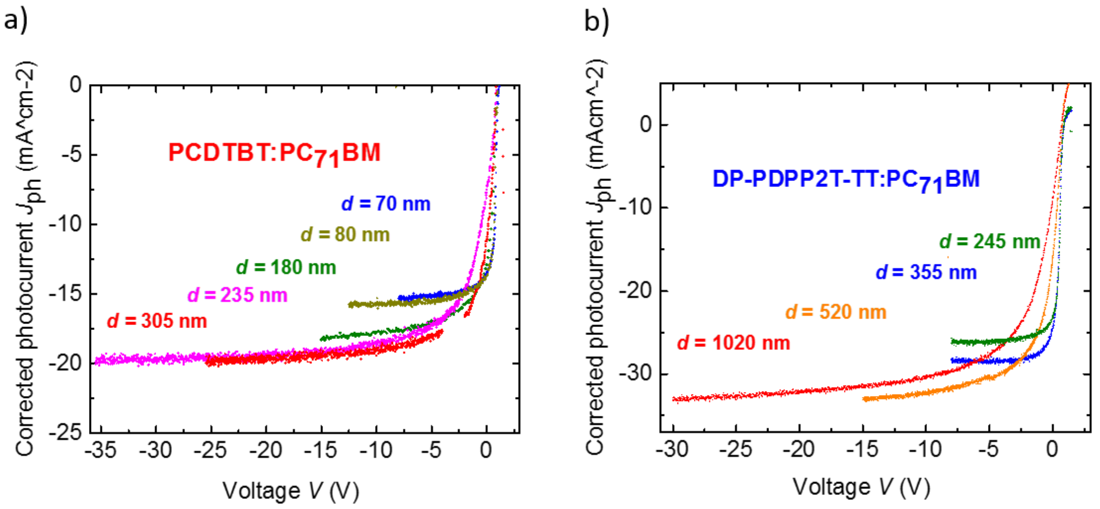

Supplementary Figure 5. Corrected photocurrent at far reverse bias. (a) and (b) shows the corrected photocurrent of PCDTBT:$PC_{71}BM$ and DT-PDPP2T-TT:$PC_{71}BM$ devices respectively with different photoactive layer thicknesses.

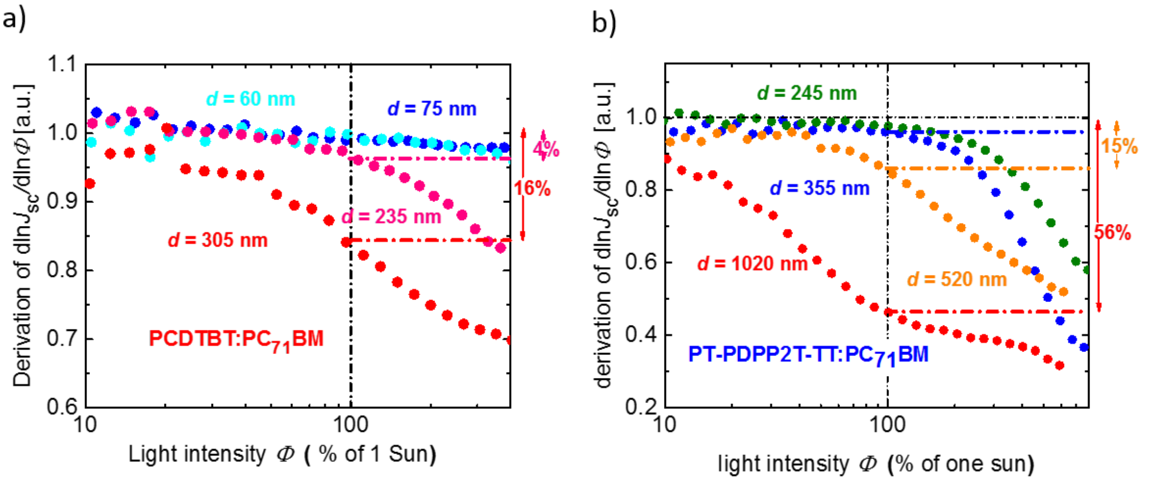

Supplementary Figure 6. Linearity analysis of short circuit current as a function of light intensity. Figure (a) and (b) show the derivation of lnJ_{sc} as function of lnΦ of PCDTBT:PC_{71}BM and DT-PDPP2T-TT:PC_{71}BM devices with different photoactive layer thicknesses, the thicker the device is, the loss of linearity of J_{sc}, indicating more bimolecular loss in thick device due to limit transport length of charge carrier.

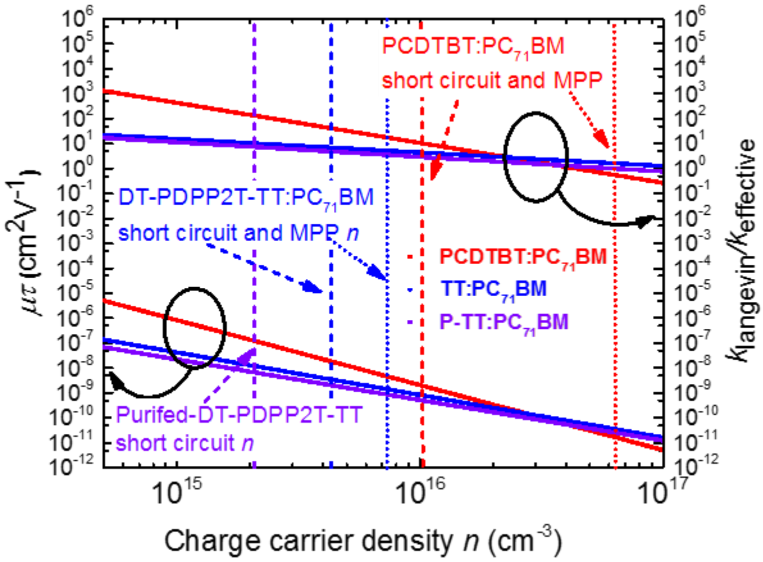

Supplementary Figure 7. Extended mobility-lifetime product and non-Langevin factor of PCDTBT:PC$_{71}$BM (red), DT-PDPP2T-TT:PC$_{71}$BM (blue) and purified-DT-PDPP2T-TT:PC$_{71}$BM (violet) devices. The dash lines represent the respective charge carrier densities measured at short circuit under 1 Sun condition, and the dotted lines represent the respective charge carrier densities measured at maximum power point (MPP) under 1 Sun conditions.

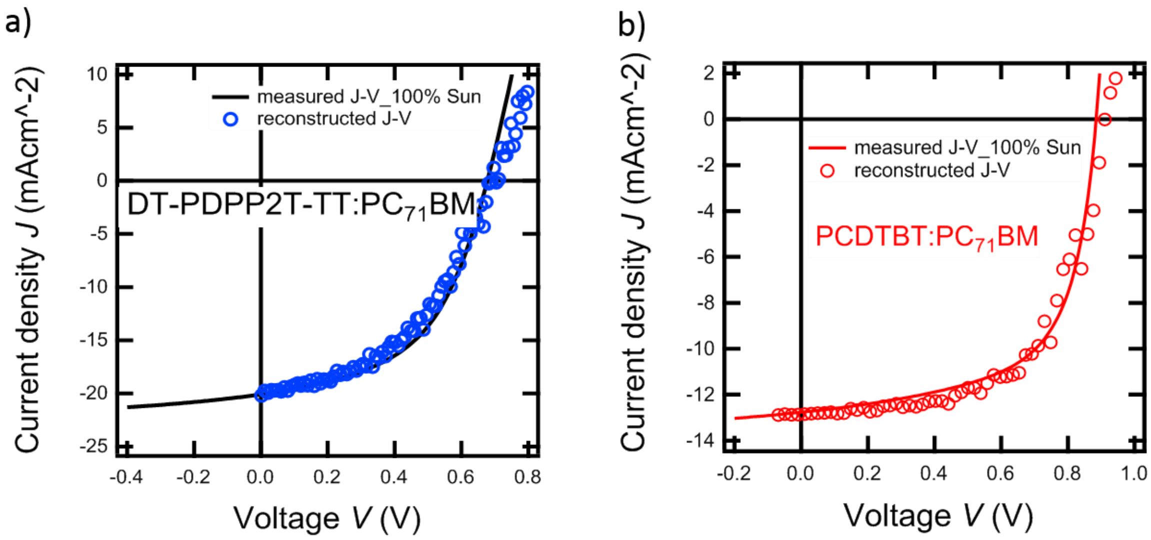

Supplementary Figure 8. *J-V* reconstruction. Figure (a) and (b) show the *J-V* reconstruction results of DT-PDPP2T-TT:$PC_{71}BM$ device and PCDTBT:$PC_{71}BM$ devices respectively measured by transient photovoltage (TPV) and charge extraction (CE). The *J-V* reconstruction model is described in Supplementary Note 2.

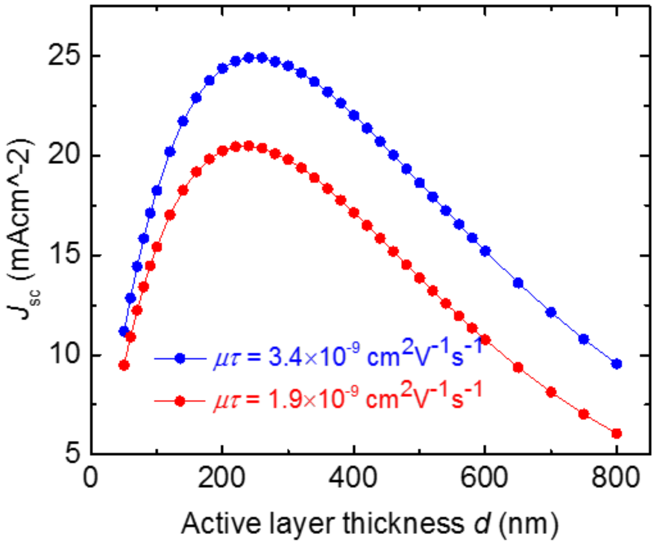

Supplementary Figure 9. Approximated J_{sc} based on the mobility-lifetime product as a function of active layer thickness. The calculation is described in Supplementary Note 3.

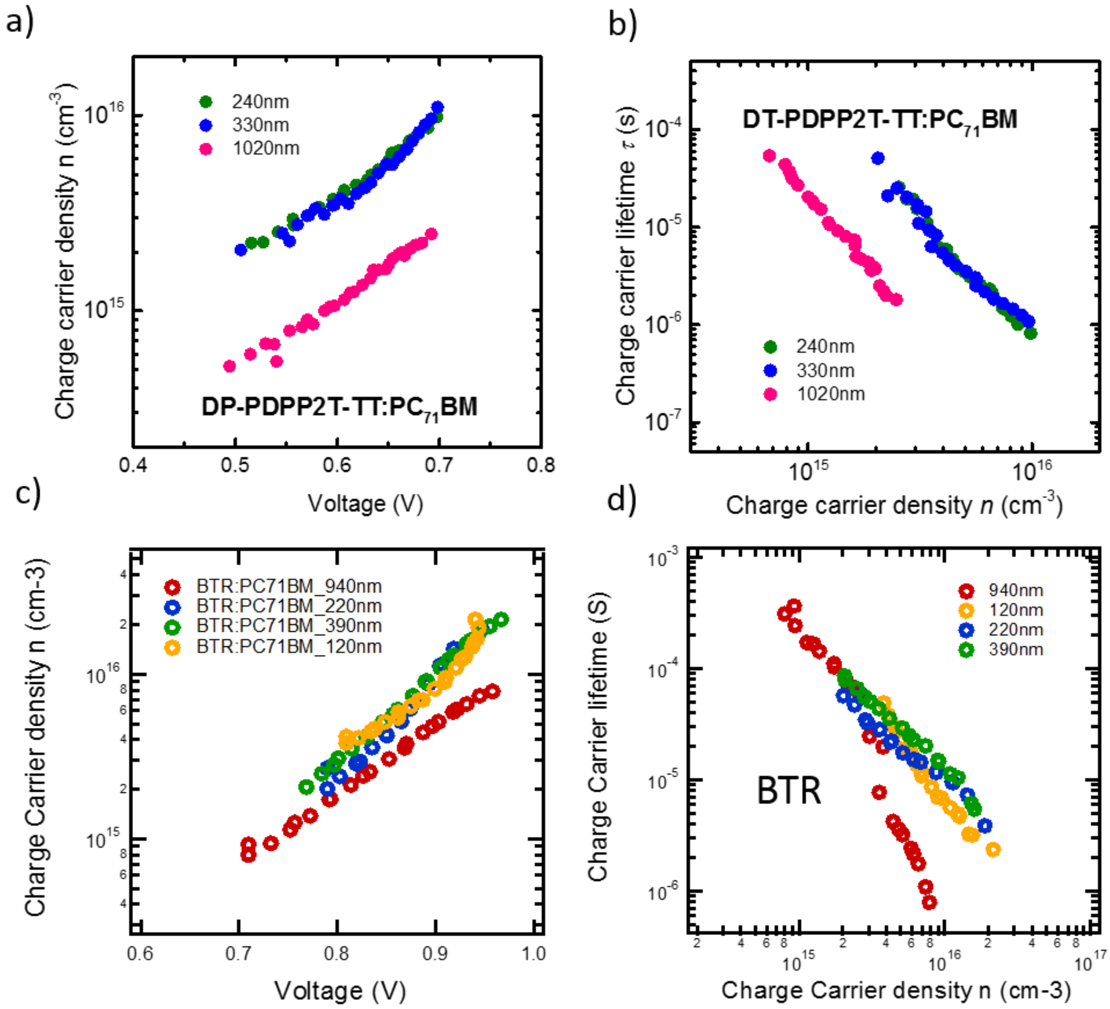

Supplementary Figure 10. Thickness-dependent energetics and recombination kinetics of DT-PDPP2T-TT:PC$_{71}$BM and BTR:PC$_{71}$BM devices. The charge carrier density and kinetics keep consistent within the effective space charge region, whereas the measured charge carrier concentration is underestimated for the thick device if the photoactive layer thickness is greater than the space charge layer width.

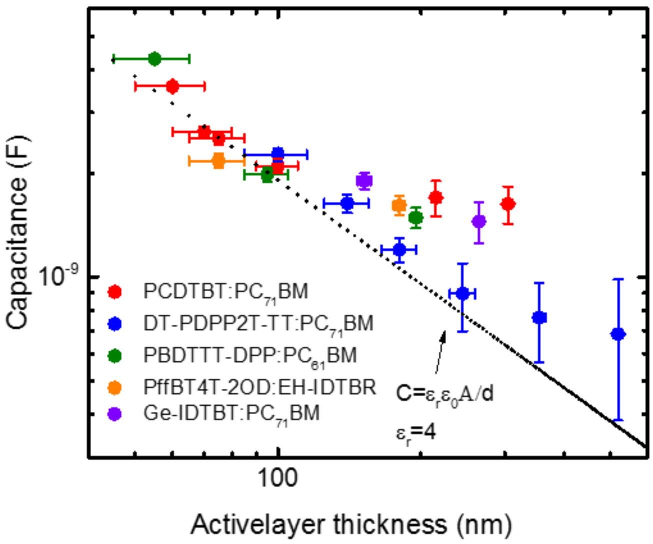

Supplementary Figure 11. Device capacitance as a function of photoactive layer thicknesses. The capacitance was measured with a small perturbation (532 nm wavelength) under a dark background condition. The total generation of charge carriers q produced by the laser perturbation is around 1×10^{-10} C, which produces a small voltage change ΔV is around 30 meV. The capacitance is determined by $C = q/\Delta V$. All devices have the same device area of 0.045 cm^2.

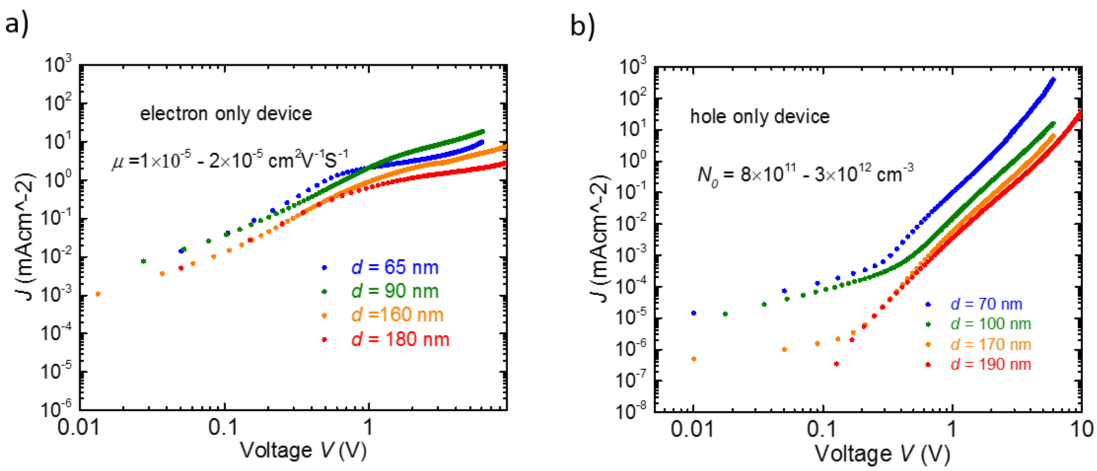

Supplementary Figure 12. Dark *J-V* measurement of PCDTBT:$PC_{71}BM$ (a) electron only and (b) hole only devices with different thickness. The hole only device has the structure of ITO/Au (80 nm)/Active layer/Au (80 nm), and electron only device has the structure of ITO/Al (80 nm)/Active layer/Al (80 nm). The electron mobility is calculated following the Mott-Gurney law, and the dark hole concentration is calculated in the ohmic regime following the work of Blom et al. in 1996.[3] However we note these data do not show ideal behaviour, possibly due to contact limitations,[4] and are therefore only included for indicative purposes.

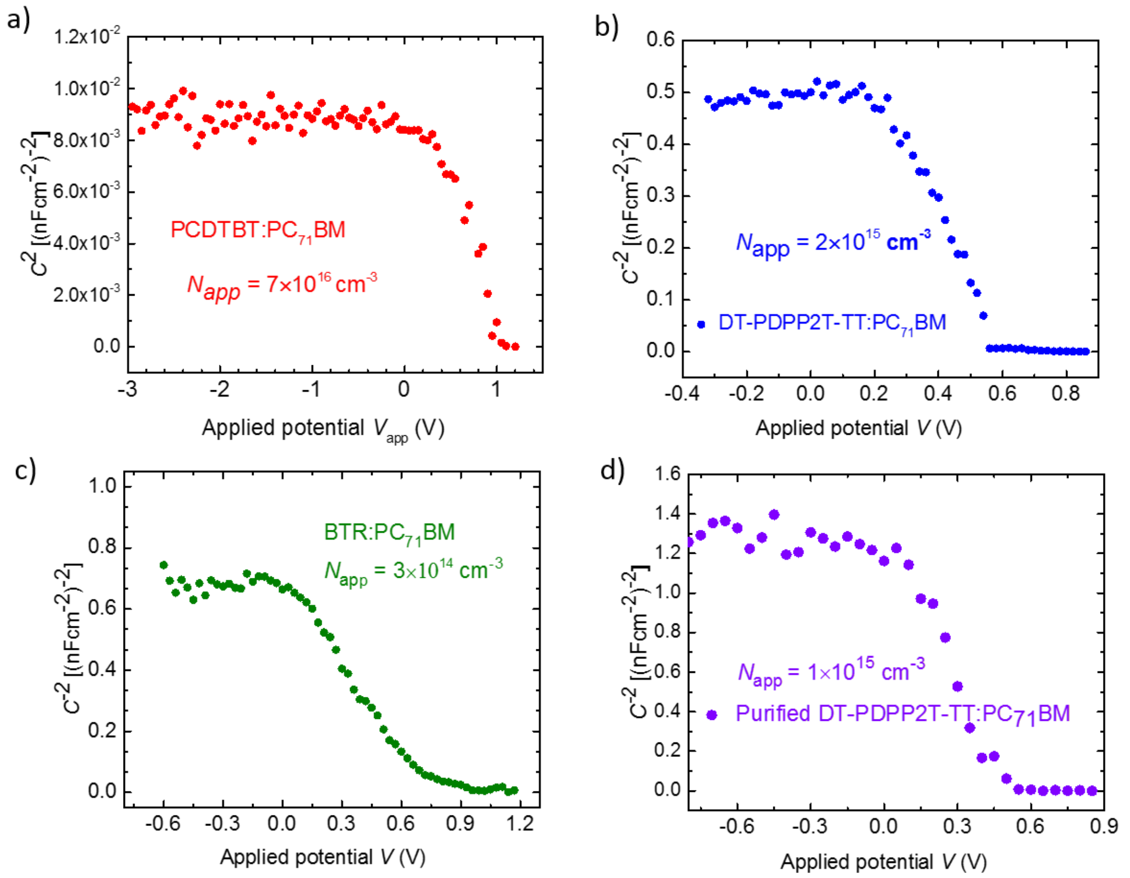

Supplementary Figure 13. Mott-Schottky analysis. The experimental results of C^{-2} as a function of applied bias are shown separately in (a) PCDTBT, (b) DT-PDPP2T-TT, (c) BTR and purified (d) DT-PDPP2T-TT blend with thick photoactive layers (all over 500 nm).

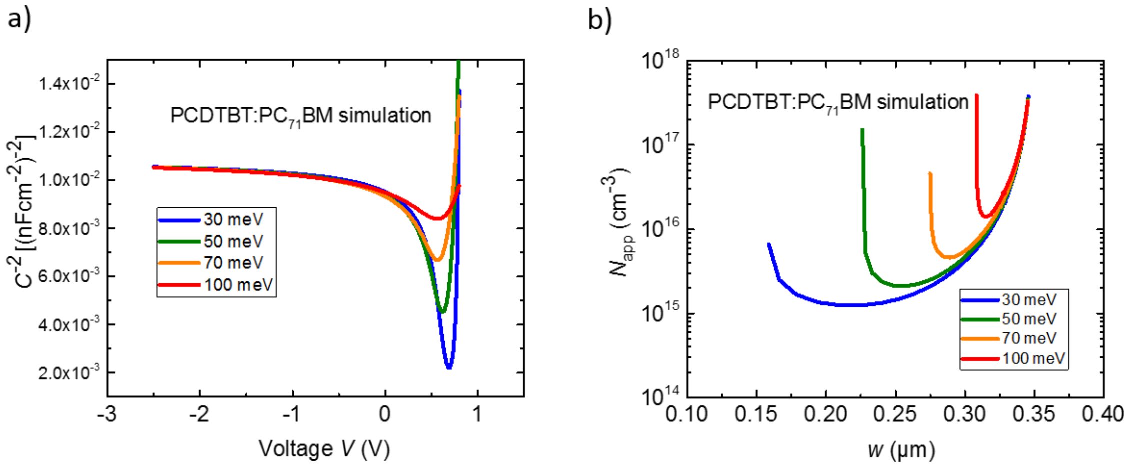

Supplementary Figure 14. Mott-Schottky simulation for PCDTBT with different tail states distributions. Figure (a) shows the C^{-2} as a function of voltage, and (b) shows the corresponding apparent doping density results.

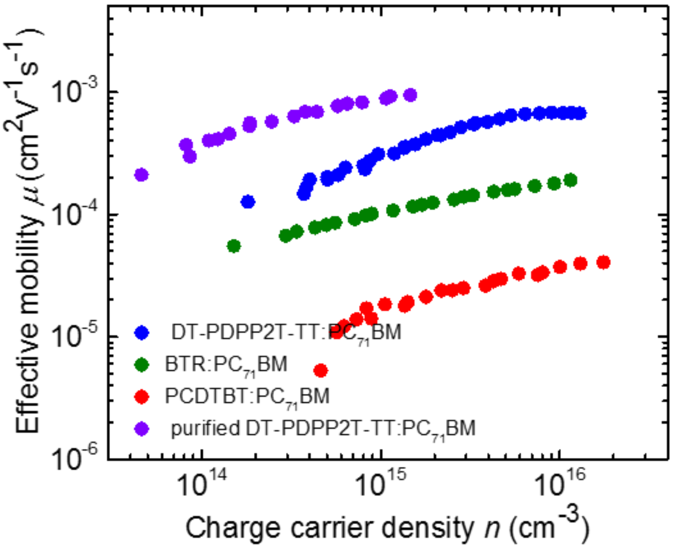

Supplementary Figure 15. Effective charge carrier mobilities. PCDTBT:PC$_{71}$BM (red) BTR:PC$_{71}$BM (green), DT-PDPP2T-TT:PC$_{71}$BM (blue) and purified DT-PDPP2T-TT:PC$_{71}$BM (violet), measured by charge extraction at short circuit.

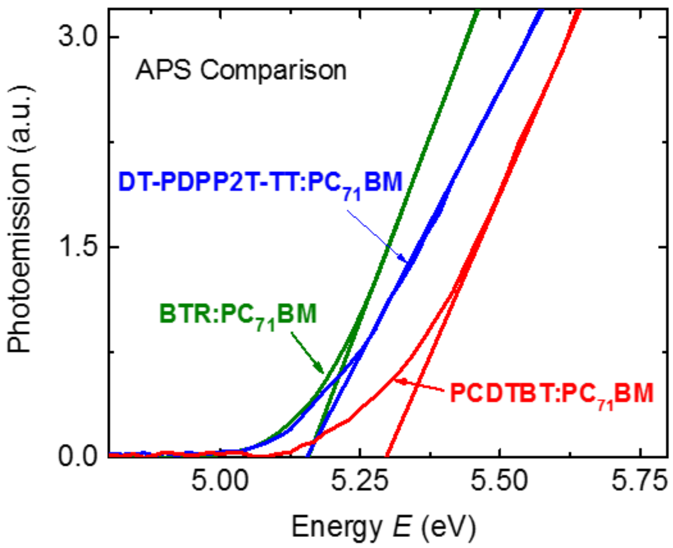

Supplementary Figure 16. Ambient Photoemission Spectroscopy results. The PCDTBT:PC_{71}BM film (red) shows the largest tail area comparing with BTR:PC_{71}BM (green) and DT-PDPP2T-TT:PC_{71}BM (blue) blend films.

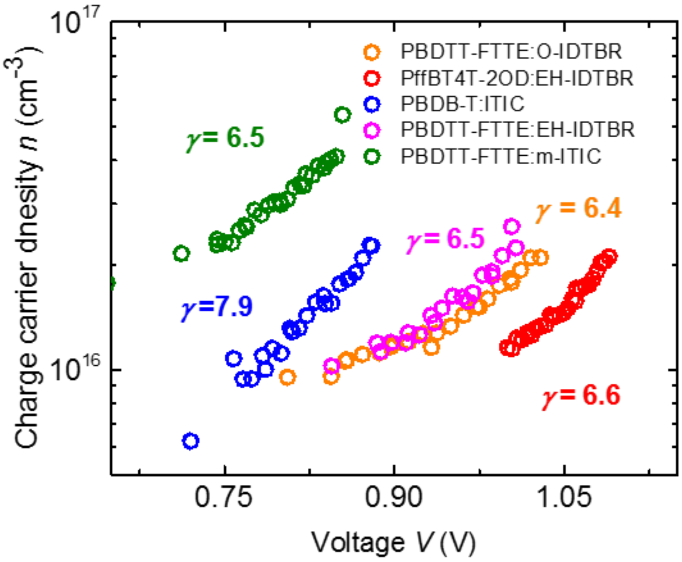

Supplementary Figure 17. Energetics of non-fullerene based PBDTT-FTTE:O-IDTBR, PBDTT-FTTE:EH-IDTBR, PBDB-T:ITIC, PBDTT-FTTE:m-ITIC and PffBT4T-2OD:EH-IDTBR devices. The charge carrier density as a function of voltage plots shows a slope at the range of 6.3 to 7.9 in these non-fullerene acceptor based device systems (the device thicknesses range between 70 to 115 nm). The slope γ is from the exponential fit $n = n_0 \exp(\gamma V_{oc})$ of the measured charge carrier density as a function of V_{oc}. The tail state slope E_{ch} derives via $E_{ch} = 1/(2\gamma)$ using the charge extraction results measured at open circuit.[5]

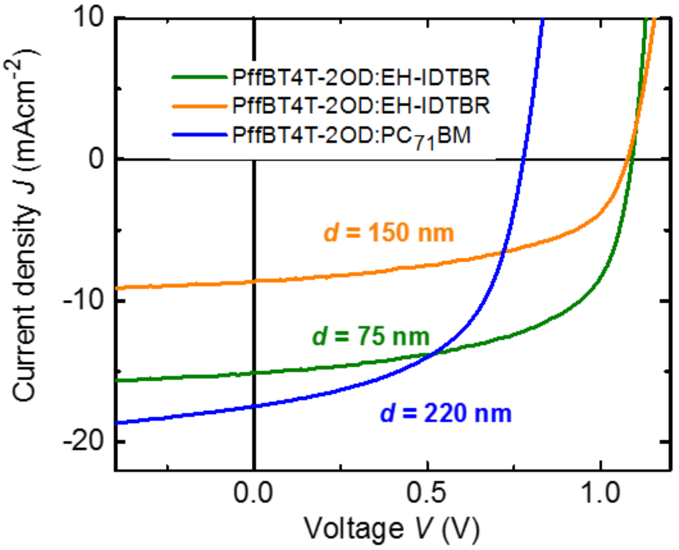

Supplementary Figure 18. *J-V* results. The *J-V* response of devices with different thickness are shown in the graph: 220 nm PffBT4T-2OD:$PC_{71}BM$ (blue), 75 nm PffBT4T-2OD:EH-IDTBR (green) and 150 nm PffBT4T-2OD:EH-IDTBR (yellow) devices measured under AM 1.5G illumination. The efficiency of thick 150 nm PffBT4T-2OD:EH-IDTBR device drops dramatically compared with optimal thin 75 nm device.

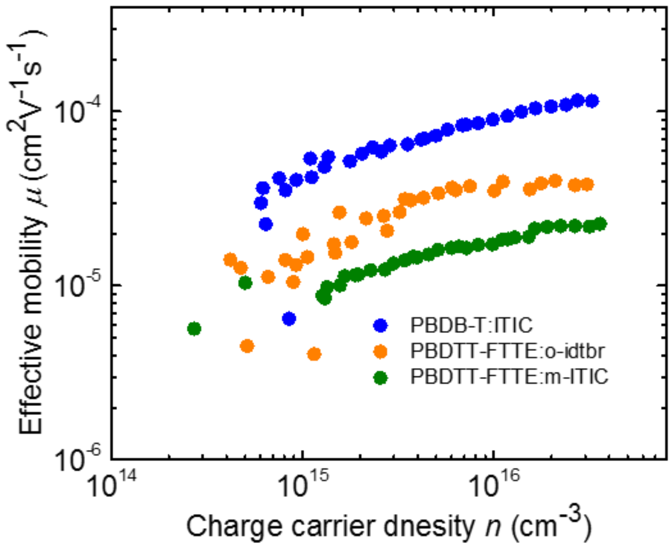

Supplementary Figure 19. Effective mobility of non-fullerene based PBDTT-FTTE:O-IDTBR, PBDB-T:ITIC and PBDTT-FTTE:m-ITIC devices.

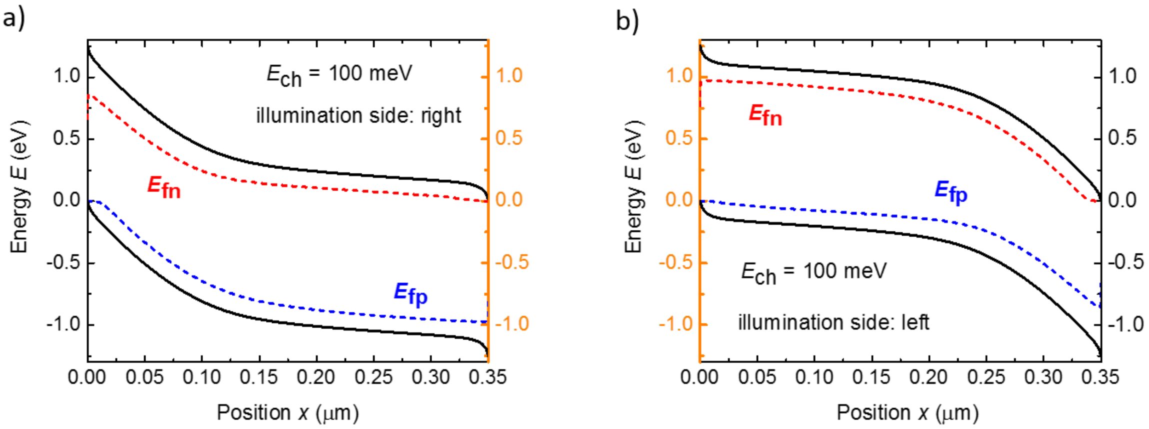

Supplementary Figure 20. Band diagram simulation of operation device with different illumination side. Figure (a) shows the simulation of the device that illuminating from the right side (the orange colour), and the formation of a space charge layer is at the left side; whereas (b) shows the same device with opposite illuminating direction (orange line and ticks side), and in this case the formation of space charge layer is at the right side. Fitting parameters can be seen in Supplementary Table 1.

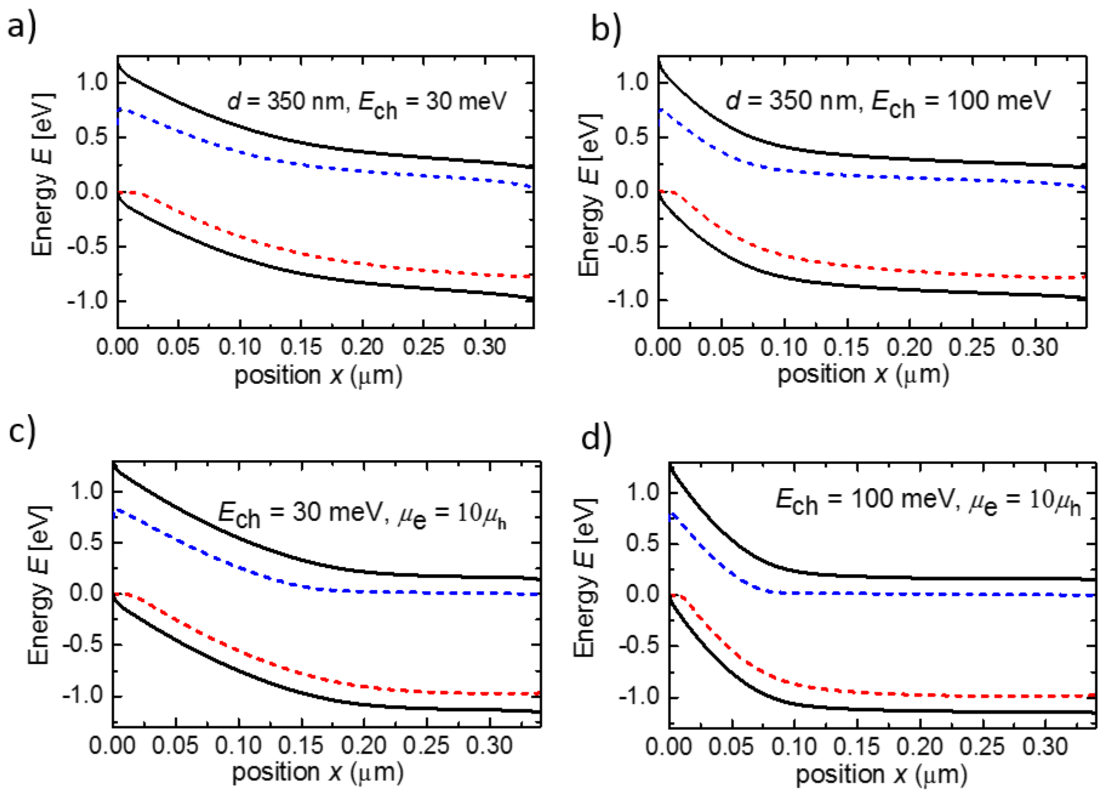

Supplementary Figure 21. Band diagrams of 350nm thick solar cells. Device with different tail states distribution and electron/hole mobilities are shown separately in (a)$E_{\mathrm{ch}} =$ 30 meV, $\mu_{\mathrm{e}} = \mu_{\mathrm{h}}$ (b), $E_{\mathrm{ch}} =$ 100 meV, $\mu_e = \mu_h$, (c) $E_{\mathrm{ch}} =$ 30 meV, $\mu_{\mathrm{e}} = 10\mu_{\mathrm{h}}$, (d) $E_{\mathrm{ch}} =$ 100 meV, $\mu_e = 10\mu_h$. Fitting parameters used are listed in Supplementary Table 1.

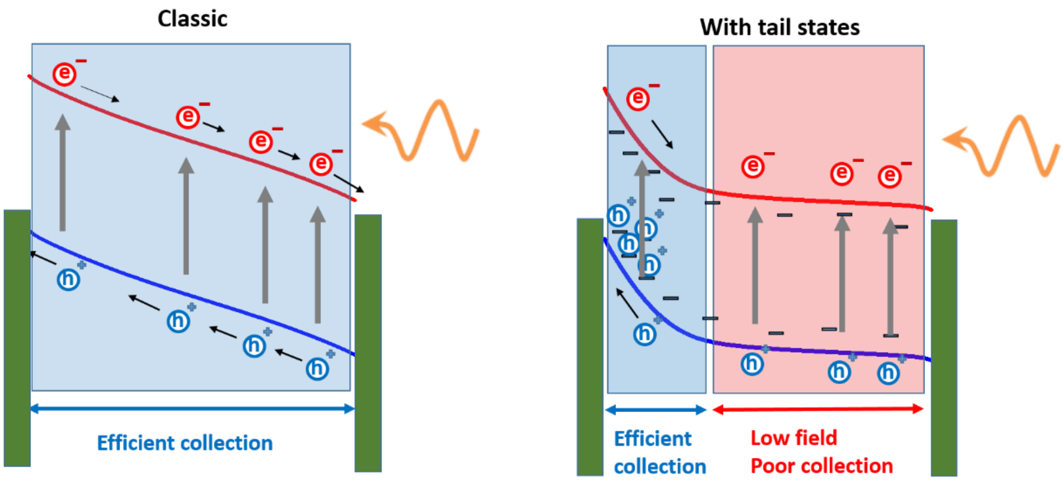

Supplementary Figure 22. Schematic illustration of charge carrier accumulation in tail states under irradiation.

Supplementary Table

Supplementary Table 1. Parameter ranges used in the simulations in Figure 4. The optical model used in the simulation is based on $\alpha(\lambda) = (A + \frac{B}{h\nu})\sqrt{h\nu - E_g}$, the absorption constant A was set to 8×10^4 $cm^{-1}eV^{0.5}$, and absorption constant B was set to 0 assuming no wavelength dependence.

**parameter**		**Figure 4**	**unit**
Thickness	d	350	nm
Effective band gap	E_g	1.25	eV
Band mobility	$\mu_{e,h}$	5×10^{-5}	$cm^2(Vs)^{-1}$
Dielectric constant	ε_r	3.5	
Effective Dos	$N_{c,v}$	10^{19}	cm^{-3}
Tail states characteristic energy	E_{ch}	$0, 100$	meV
Total tail states density	N_t	$0, 2\times10^{17}$	cm^{-3}
doping	N_d	$0, 3\times10^{16}$	cm^{-3}
Capture coefficient	$\beta_{e.h}$	2.5×10^{-12}	cm^3s^{-1}

Supplementary Notes

Supplementary Note 1. Biomolecular loss calculation at short circuit.

The current loss via non-geminate bimolecular recombination is determined via $J_{\mathrm{NG}} = ed\bar{R}$ where the $\bar{R}$ represent the spatially averaged recombination rate, we note that this analysis is typically based on homogeneous field cross the whole photoactive layer, which means the d used is always equal to the photoactive layer. The $\bar{R}$ can be approximated by measuring the product of effective bimolecular recombination coefficient k_{bi} and the square of charge carrier density n^2: $\bar{R} = edk_{\mathrm{bi}}n^2$ (assume the hole carrier density equal to the electron carrier density). The current loss $J_{\mathrm{NG}}(d)$ as a function of photoactive layer thickness is therefore can be calculated for both systems. Under short circuit condition, due to efficient charge extraction because of relatively strong internal field present within the junction, the charge carrier density is therefore rather small compare with low internal field conditions such as open circuit, then it is apparent that the $J_{\mathrm{NG\_loss}}$ at short circuit is small. Here we have to note that even for fast non-geminate recombination system such as sample blends PCDTBT:PC_{71}BM and DT-PDPP2T-TT:PC_{71}BM we provide here, in which the effective recombination coefficient is as high as 10^{-11} cm^3s^{-1} (compare with reported slow recombination system of 10^{-13} cm^3s^{-1}, or known as non-langevin type of recombination such as P3HT system). Those current losses of PCDTBT and DT-PDPP2T-TT thick devices up to 400nm are as small as 1.75 $mAcm^{-2}$ and 1.80 $mAcm^{-2}$ with the charge carrier density 8.5×10^{15} cm^{-3} and 4.5×10^{15} cm^{-3} respectively. However, the gap between J_{sc} and J_{max} of PCDTBT 400nm device is as large as 9 $mAcm^{-2}$. This current added back from bimolecular loss $J_{\mathrm{sc}} + J_{\mathrm{NG\_loss}}$ failed to fill the gap between J_{max} from > 100 nm for PCDTBT system, and failed from > 350nm for DT-PDPP2T-TT system. To double confirm the value of current loss via non-geminate bimolecular recombination to avoid the case of non-fully charge extraction via CE, photocurrent linearity was taken. This bimolecular current loss at high light level is

quantified via $J_{\text{linearityloss}} = 10 * J_{10\%sun} - J_{100\%sun}$, where $J_{100\%Sun}$ is the J_{sc} measured under 10% of 1sun equivalent condition and $J_{100\%Sun}$ is the J_{sc} measured under 100% of 1sun equivalent illumination. The linearity of photocurrent as a function of light intensities has been plotted in Supplementary Figure 6, at low light condition, due to low charge carrier density, the bimolecular recombination is negligible, whereas at higher light condition, the photo-generated charge carrier density increase, the bimolecular recombination occurs, and therefore the ratio of the photocurrent with light intensity ($dlnJ_{sc}/dln\Phi$) starts losing linearity. In our cases, at 10% of 1sun equivalent illumination, the linearity is unchanged, which is assumed no significant bimolecular recombination occurs. The results calculated through linearity analysis are similar to calculation measured via CE and TPV showed in Figure 2, the slightly higher value in PCDTBT case indicates it might have more traps that charge carriers are not fully extracted from this device via CE technique.

Supplementary Note 2. *J-V* reconstruction.

The current density can be described by the continuity equation at every operating point across the voltage range:

$$\frac{1}{q}\frac{dJ}{dx} + G - R = 0 \qquad 1$$

J is current density, *q* is the elementary charge, *x* is the spatial location of the device, *G* is generation rate in volume, *R* is recombination rate.

Using the charge extraction across the *J-V*, the charge carrier density at different operating bias can be assessed. Therefore, the integration of Eq.1 across the active layer thickness we can obtain the *J-V* response:

$$J(V) = -qG(V)d + qdR(V) \qquad 2$$

If the generation is field independent, $G(V)$ is independent to bias therefore is a constant, here we use $qG(V)d = J_{ph}^{saturated}$. $qdR(V)$ is the recombination flux at different bias conditions,

where the recombination rate $R(V)$ is defined as $R(V)=k_{\mathrm{bi}}(\mathrm{n}(V))^2$, k_{bi} is effective bimolecular recombination coefficient calculated by TPV/CE results measured at open circuit.

Supplementary Note 3. Photocurrent calculation.

The photocurrent is simulated via the Hecht equation $J_{\mathrm{sc}} = 2q\bar{G}\mu\tau\frac{V_{\mathrm{bi}}}{d}(1-\exp(-\frac{d^2}{2\mu\tau V_{\mathrm{bi}}}))$,[1,2] the photocurrent collected at short circuit depends on the mobility-lifetime (μτ) product. At short circuit, the internal field equals to the build-in potential (V_{bi}). The $\mu\tau$ used (1.9×10^{-9} $cm^2V^{-1}s^{-1}$ and 3.4×10^{-9} $cm^2V^{-1}s^{-1}$) in this the graph is based on the measurements of PCDTBT:$PC_{71}BM$ and DT-PDPP2T-TT:$PC_{71}BM$ devices respectively.

Supplementary Note 4. Evaluation of photoactive layer doping density. It has previously been suggested that inadvertent doping of the photoactive layer can result, for thick devices, in a space charge layer width *w* less than the photoactive layer width.[15,19,20] Device band diagram simulations to illustrate this effect are shown in Figure 4, with Figures 6a and 6b illustrating undoped and doped devices respectively. For the doped device, it is apparent that the doping density of 3×10^{16} cm^{-3} used in these simulations results in a space charge width of around 100 nm. Based on this model, calculations of space charge layer width determined by doping density indicate that our experimental determination, from Figure 3, of space charge layer widths of circa 80 and 400 nm for PCDTBT and DT-PDPP2T-TT blends would require dark doping densities of 5×10^{16} cm^{-3} and 2.4×10^{15} cm^{-3}, respectively. We employed work function measurements to determine whether these blends exhibit sufficient inadvertent dark doping to generate such charge densities. For both PCDTBT:$PC_{71}BM$ and DT-PDPP2T-TT:$PC_{71}BM$, the bulk work function of the blends was determined to lie slightly below the middle of the gap – i.e., both blends seem slightly p-type doped as shown in Figure 5a, with the DT-PDPP2T-TT:$PC_{71}BM$ seems to be more doped than the PCDTBT:$PC_{71}BM$. Assuming an effective valence band edge density of states of 10^{20} cm^{-3} (an upper estimate), the energy difference

measured between the Fermi level E_F and valence band edge E_V of DT-PDPP2T-TT:$PC_{71}BM$ of 0.3 eV corresponds to an approximate doping density of 8×10^{13} cm^{-3}. The equivalent analysis for PCDTBT:$PC_{71}BM$ gives doping density of 2×10^{12} cm^{-3}. It can be concluded that both DT-PDPP2T-TT:$PC_{71}BM$ and PCDTBT:$PC_{71}BM$, the absolute doping density values are unable to explain the space charge layer widths observed for these devices. We also observed a change in band bending direction of PCDTBT:$PC_{71}BM$ film on different substrates as shown in Figure 5b, which again suggests no significant dark doping in the blend. We thus conclude that the smaller space charge layer width of PCDTBT system is not caused by inadvertent dopants. The low concentration of dopants of the PCDTBT system was further indicated by *J-V* responses in the dark of electron and hole only devices, as shown in Supplementary Figure 12. The electron-only devices present an apparent space charge limited current (SCLC) region which gives electron mobility in the range of around10^{-5} $cm^2V^{-1}s^{-1}$, in agreement with the mobility measured via charge extraction at short circuit. In hole-only devices, there is an Ohmic region shown at low voltages. Analysis of this region using $J = qp\mu_p V/d$, yields a charge carrier density of 3×10^{12} cm^{-3}, consistent with our work function data, and again confirming that the space charge width limitations determined above do not result from high dark doping of these materials.

Supplementary References

1. Crandall, R. S. Transport in hydrogenated amorphous silicon p - i - n solar cells. *J. Appl. Phys.* **53,** 3350–3352 (1982).
2. Hecht, K. Zum Mechanismus des lichtelektrischen Prim rstromes in isolierenden Kristallen. *Zeitschrift Phys.* **77,** 235–245 (1932).
3. Blom, P. W. M., de Jong, M. J. M. & Vleggaar, J. J. M. Electron and hole transport in poly(p - phenylene vinylene) devices. *Appl. Phys. Lett.* **68,** 3308–3310 (1996).
4. Röhr, J. A., Moia, D., Haque, S. A., Kirchartz, T. & Nelson, J. Exploring the validity and limitations of the Mott–Gurney law for charge-carrier mobility determination of

semiconducting thin-films. *J. Phys. Condens. Matter* **30,** 105901 (2018).
5. Kirchartz, T. & Nelson, J. Meaning of reaction orders in polymer:fullerene solar cells. *Phys. Rev. B* **86,** 165201 (2012).
